# Factors associated with failure to start consolidation durvalumab after definitive chemoradiation for locally advanced NSCLC

**DOI:** 10.3389/fonc.2023.1217424

**Published:** 2023-07-05

**Authors:** Christian Wilhelm Langberg, Henrik Horndalsveen, Åslaug Helland, Vilde Drageset Haakensen

**Affiliations:** ^1^ Faculty of Medicine, University of Oslo, Oslo, Norway; ^2^ Department of Oncology, Oslo University Hospital, Oslo, Norway; ^3^ Department of Cancer Genetics, Institute for Cancer Research, Oslo University Hospital, Oslo, Norway; ^4^ Institute for Clinical Medicine, Faculty of Medicine, University of Oslo, Oslo, Norway

**Keywords:** non-small cell lung cancer, chemoradiotherapy, toxicity, durvalumab, prehabilitation, personalized treatment

## Abstract

**Introduction:**

The introduction of consolidation immunotherapy after chemoradiotherapy has improved outcome for patients with locally advanced non-small cell lung cancer. However, not all patients receive this treatment. This study identifies factors associated with failure to start durvalumab as consolidation therapy with the aim of optimizing treatment, supportive care and prehabilitation to ensure that more patients complete the planned treatment.

**Materials and methods:**

Patients from two clinical trials and a named patient use program, were included in this study. All patients received platinum-doublet chemotherapy concomitant with radiotherapy to a total dose of 60-66 gray. Patient characteristics, cancer treatment, toxicity, performance status and laboratory data before and after chemoradiotherapy were recorded and patients who never started durvalumab were compared with those who did.

**Results:**

A total of 101 patients were included, of which 83 started treatments with durvalumab after chemoradiotherapy. The 18 patients who did not start durvalumab had significantly higher lactate dehydrogenase at baseline and a worse performance status, cumulative toxicity and higher c-reactive protein after completed chemoradiotherapy. Data also suggest that pre-treatment diabetes and reduced hemoglobin and/or diffusion capacity of the lungs for carbon monoxide contribute to the risk of treatment abruption.

**Conclusion:**

Treatment plan disruption rate was 18%. Systemic inflammation and performance status were associated with failure to receive durvalumab after chemoradiation. Further studies are needed to confirm findings and prospective trials should investigate whether prehabilitation and supportive treatment could help more patients finishing the planned treatment.

**Clinical Trial Registration:**

clinicaltrials.gov, identifier NCT03798535; NCT04392505.

## 1 Introduction

Non-small cell lung cancer (NSCLC) is a common disease carrying a poor prognosis. About 20% of these patients present with locally advanced disease, a heterogenous group where not all cases are suited for intensive and curatively intended treatment. Performance status, age, tumor size and prognostic factors as weight loss may influence the choice of therapy (1, 2). However, the majority of patients with stage III disease can potentially be treated with curative intent, either by surgery or by using radiotherapy to 60-66 gray (Gy) with concomitant platinum-doublet chemotherapy resulting in a 5-year overall survival of 15-30% (3). The PACIFIC trial showed that one year of durvalumab (monoclonal PD-L1 antibody) after completion of chemoradiotherapy (CRT), prolonged progression free survival (PFS) and overall survival (OS) of these patients (3, 4). While the Food and Drug Administration (FDA) approved durvalumab for all patients with no disease progression after completed CRT, European Medicines Agency (EMA) has approved the treatment for patients with programmed cell death ligand 1 (PD-L1) positive tumors only. Despite guidelines, some patients never start the planned consolidation immunotherapy. It may therefore be of importance to elucidate factors that may influence the initiation of durvalumab in this setting.

CRT followed by immunotherapy may offer a challenging and potentially harmful path to recovery for vulnerable NSCLC patients. Previous studies with CRT alone reported an incidence of ≥ grade 3 toxicity of about 20% (5) and that CRT-induced pneumonitis was an important toxicity impacting quality of life (6). Pneumonitis ≥ grade 3 has been reported in up to 10% of patients receiving CRT alone (7). A recent study with CRT + ipilimumab and nivolumab was stopped due to pulmonary toxicity which limited opportunities for improved outcomes (8). In the PACIFIC trial patients were included after CRT if they had not progressed and were fit enough to start durvalumab. It is therefore unknown how many patients who received CRT only and failed to start durvalumab. Our study investigates factors that may influence the initiation of immunotherapy after completed CRT. By identifying such factors, supportive measures may be tailored to secure the optimal treatment for these patients in the future.

## 2 Material and methods

### 2.1 Study population and data collection

This is a retrospective study of patients with locally advanced NSCLC treated with CRT with curative intent, eligible for durvalumab.

Data for this study was retrieved from patients included in two clinical trials: PACIFIC-R (First Real-world Data on Unresectable Stage III NSCLC Patients Treated With Durvalumab After Chemoradiotherapy) (clinicaltrials.gov: NCT03798535) and DART (Durvalumab After chemoradiotherapy for NSCLC (clinicaltrials.gov: NCT04392505), as well as a named patient use program for durvalumab.

The PACIFIC-R trial is a retrospective international multicenter study that collects information from patients included in a named patient use program for durvalumab, following the presentation of results from the PACIFIC trial at ESMO in 2017. Patients accepted in the program, but who did not receive durvalumab were not included in the PACIFIC-R trial, but are included in the current analyses.

The DART trial is a biomarker study which includes patients with locally advanced NSCLC treated with CRT, followed by one year of durvalumab. Patients that received CRT, but were not treated with durvalumab, are excluded from the DART primary end-point analyses but are included in the current study.

Patients were included from 2017 until 2022. From the PACIFIC-R study, only patients recruited at Oslo University Hospital were included in the current analyses. Patients in the DART study were recruited from Oslo University Hospital, Stavanger University Hospital, Haukeland University Hospital, University Hospital of North-Norway, St. Olavs Hospital, Tampere University Hospital, Oulu University Hospital, North Estonia Medical Centre, Vilnius University Hospital and Turku University Hospital.

Data in this study was collected from study databases (electronic case report forms), patient journals, radiation dose-plans and treatment records. The following parameters were recorded: patient demographics and clinical characteristics such as sex, age, Easter Cooperative Oncology Group (ECOG) performance status, tumor histology, PD-L1 status, tumor stage according to the TNM classification of malignant tumors, 8th edition (9), medical history, smoking status, lung function, laboratory analyses and cancer treatment received (including chemotherapy, radiotherapy and immunotherapy, if administrated). Adverse events occurring within a period of three months after completed CRT were registered, using the Common Terminology Criteria for Adverse Events (CTCAE) version 5.0 (10).

### 2.2 Chemoradiotherapy

Radiotherapy was planned after delineation of gross tumor volume (GTV) of tumor and involved lymph nodes on a free-breathing CT and expanded to include breathing motion assessed by a 4DCT (iGTV). A 5 mm margin was added to the iGTV to create the clinical target volume (CTV) which was cropped for organs at risk e.g. bone and large vessels. A 5-8 mm margin was used to create the planning target volume (PTV). Treatment planning was performed in Varian Eclipse v13.6 or RayStation v5 using either volumetric-modulated arc therapy (VMAT) or intensity-modulated radiotherapy (IMRT). Image-guided radiotherapy (IGRT) was delivered with daily cone-beam CT (CBCT) prior to treatment (11). All patients received a total dose of 60-66 Gy in 2 Gy fractions. Dose constraints to organs at risk were as follows: mean lung dose (MLD) < 20 Gy, percentage of normal lung volume that received 20 Gy or more (lung V20) < 35 Gy, mean esophagus dose < 34 Gy and the dose to one cm^3^ of the esophagus (D1ccm) < 68 Gy. Dose planning was performed according to International Committee for Radiological Units (ICRU) (12) and local dose volume constraints.

Platinum-based chemotherapy was administered concomitantly with radiotherapy every three weeks. In accordance with national guidelines and standard of care in Norway, platinum-doublet chemotherapy consisted of either cisplatin 75 mg/m2 IV or carboplatin area under the curve (AUC) 6 IV day 1 combined with etoposide 100 mg/m2 IV day 1-3 or vinorelbine 50 mg PO three days a week. Dose reductions were performed for some patients based on clinical evaluation.

### 2.3 Statistical analysis

Statistical analyses including descriptive analysis, Student’s t-tests, Chi-squared tests, Fisher’s exact tests, Wilcoxon signed rank tests and Mann Whitney u tests were used when appropriate and multivariate analysis with logistical regression was also performed. All tests were two-sided, and a p-value ≤ 0.05 was considered statistically significant. Statistical analyses were performed using IBM SPSS Statistics, version 29.0.0.0.

### 2.4 Ethics

This study was approved by the regional ethics committee in the South-Eastern Health Region, Norway (22/426980) and all patients signed informed consent for one of the clinical trials or accepted data collection as part of a named patient use program.

## 3 Results

### 3.1 Patient characteristics

**Figure 1:**
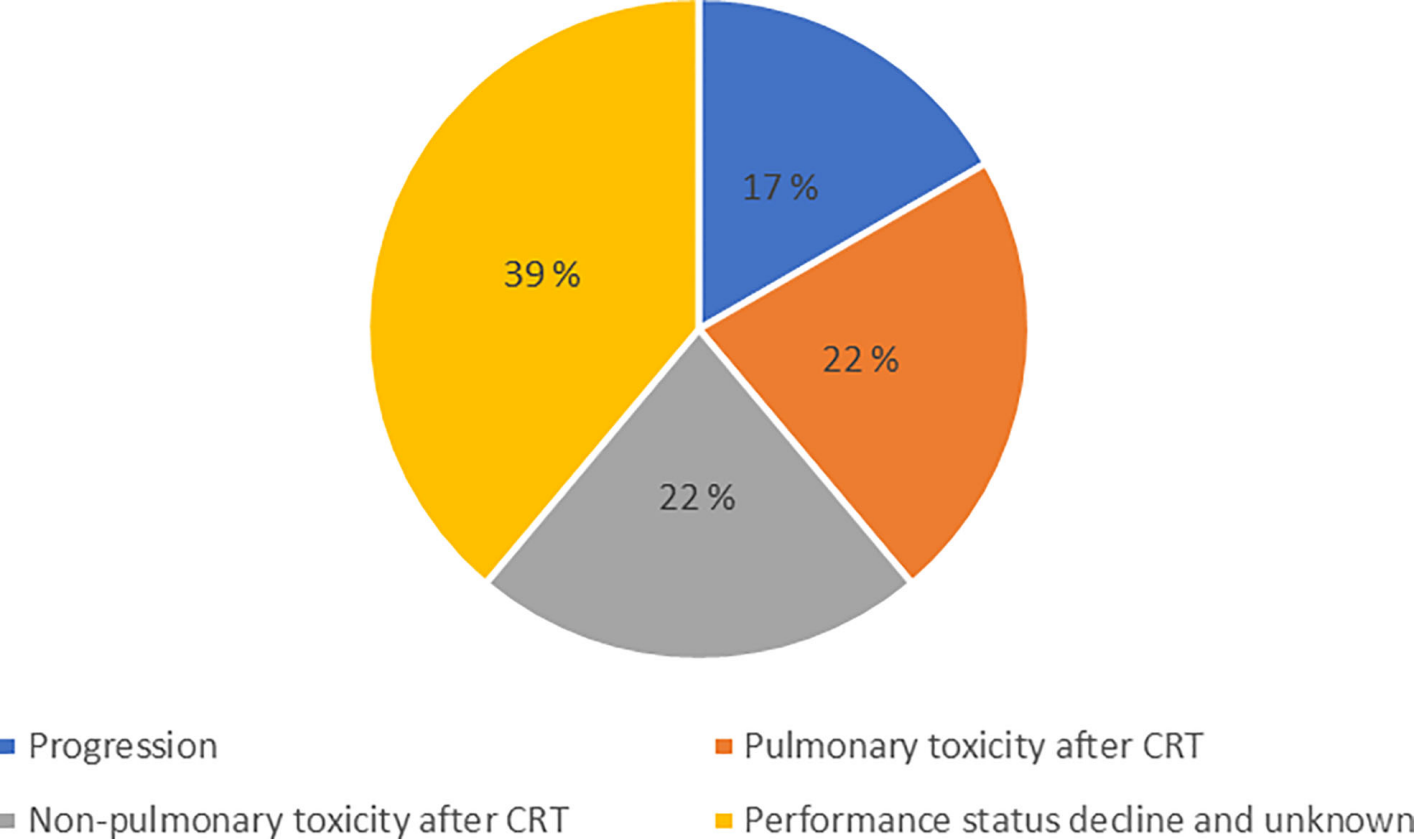
Disruption of treatment plan. The reasons why patients eligible for consolidation durvalumab did not receive this treatment.

A total of 101 patients were included, 18 patients received CRT only while 83 patients received CRT and durvalumab. The most common reasons for not starting durvalumab were toxicity (44%), poor performance status (39%) and disease progression (17%) (**Figure 1**). The median time from completed CRT to start of durvalumab was 31 days. A total of 10 patients were excluded from the study due to: screening failure (brain metastasis and wrong staging), withdrawal of consent, not completing CRT (one patient only got radiotherapy due to medical comorbidity, one never got CRT and one only received a total of 24 Gy) and unknown cause (**Supplementary Figure 1**).

Patient demographics, performance status, tumor stage, histology, PD-L1 status, smoking and comorbidities are shown in **Table 1**. Seven patients were included despite being diagnosed with stage IIB disease as they were considered candidates for CRT plus durvalumab and not surgery or stereotactic radiotherapy due to local tumor invasion or medical comorbidities. No significant statistical differences in baseline values were found between the patients who received CRT only, and those who received CRT followed by durvalumab. The patients who received durvalumab seemed to have more adenocarcinomas and less low differentiated tumors, but this difference was not statistically significant.

**Table 1:** Baseline patient characteristics.

Baseline characteristics	CRT only (n = 18)	CRT + D (n = 83)	All patients (n = 101)	P-value
Age, years				0.59
Median (range)	68 (57-78)	68 (36-85)	68 (36-85)	
Sex, n (%)				0.59
Male	12 (67)	47 (57)	59 (58)	
Female	6 (33)	36 (43)	42 (42)	
ECOG status before CRT, n (%)				0.60
0	8 (44)	39 (47)	47 (47)	
1	10 (56)	35 (42)	45 (45)	
Unknown	0 (0)	9 (11)	9 (9)	
Disease stage, n (%)				0.65
IIB	2 (11)	5 (6)	7 (7)	
IIIA	10 (56)	42 (51)	52 (51)	
IIIB	6 (33)	30 (36)	36 (36)	
IIIC	0 (0)	6 (7)	6 (6)	
Histologic subtype, n (%)				0.06
Squamous cell carcinoma	9 (50)	44 (53)	53 (52)	
Adenocarcinoma	6 (33)	37 (45)	43 (43)	
Other*	3 (17)	2 (2)	5 (5)	
PD-L1 status, n (%)**				0.77
Positive	11 (61)	57 (69)	68 (67)	
Negative	6 (33)	24 (29)	30 (30)	
Unknown	1 (6)	2 (2)	3 (3)	
Smoking status, n (%)				0.49
Previously smoked daily	12 (67)	61 (73)	73 (73)	
Daily smoker	6 (33)	17 (20)	23 (23)	
Never smoked	0 (0)	5 (6)	5 (5)	
Medical history, n (%)				
Lung disease	10 (56)	28 (34)	38 (38)	0.11
Heart disease	12 (67)	41 (49)	53 (52)	0.21
Diabetes	1 (6)	16 (19)	17 (17)	0.30
Other cancer	3 (17)	20 (24)	23 (23)	0.76
Autoimmune disease	1 (6)	9 (11)	10 (10)	0.69
Other	6 (33)	18 (22)	24 (24)	0.36
Lung function before CRT (mean)				
FEV1, l	1.9	2.2	2.2	0.15
FEV1, %	69.7	78.3	76.7	0.17
DLCO, %	60.2	69.2	67.3	0.07

*Other histological subtypes were low differentiated carcinomas. ** PD-L1: limit for positive PD-L1 status was appointed to above or equal to 1%.

CRT + D: Patients receiving durvalumab after CRT, FEV1, forced expiratory volume in 1 second; DLCO, diffusion capacity of the lungs for carbon monoxide.

Lung function tests showed no differences between the two groups regarding forced expiratory volume in 1 second (FEV1). However, the group who only received CRT, had borderline worse diffusion capacity of the lungs for carbon monoxide (DLCO) (p-value = 0.07) (**Table 1**).

### 3.2 Chemoradiotherapy

An overview of the chemotherapy regimens and radiotherapy doses given is shown in **Table 2**. No statistically significant difference in CRT data was detected between the two study groups.

**Table 2:** Chemoradiotherapy data.

Chemoradiotherapy data	CRT only (n = 18)	CRT + D (n = 83)	All patients (n = 101)	P-value
Carboplatin, n (%)	5 (28)	32 (39)	37 (37)	0.43
Cisplatin, n (%)	11 (61)	60 (72)	71 (70)	0.4
Etoposide, n (%)	14 (78)	66 (80)	80 (79)	1
Vinorelbine, n (%)	2 (11)	17 (20)	19 (19)	0.51
Total dose, Gy, mean, (range)	64.4 (60-66)	64.9 (60-66)	64.9	0.43
PTV, cm^3^, mean, (range)	436.5 (182-1197)	425.4 (70-979)	427.3	0.88
MLD, Gy, mean, (range)	14.9 (8-19)	12.4 (3-21)	12.9	0.054
Lung V_20_, %, mean, (range)	28.2 (21-35)	21.4 (5-35)	22.5	0.005
Mean heart dose, Gy, mean, (range)	12.5 (1-20)	8.3 (0-33)	9.0	0.06
Mean oesophagus dose, Gy, mean, (range)	23.2 (7-38)	19.7 (4-61)	20.3	0.25

CRT + D: Patients receiving durvalumab after CRT. PTV, planning target volume; MLD, mean lung dose; Lung V20, the percentage of normal lung volume receiving at least 20 Gy.

### 3.3 Toxicity

The patients’ ECOG performance status at baseline was 0-1 by inclusion criteria. After CRT, the group of patients who received CRT only had a significantly higher ECOG status compared to the group that received consolidation durvalumab, as presented in **Table 3**.

**Table 3:** ECOG performance status and toxicity of ≥ grade 3 (CTCAE 5.0) after CRT (no grade 4 was observed).

ECOG status after CRT, n (%)	CRT only (n = 16)	CRT + D (n= 77)	All patients (n = 93)	P-value
ECOG 0	5 (31)	12 (16)	17 (18)	0.02
ECOG 1	6 (38)	53 (69)	59 (63)	
ECOG 2	3 (19)	11 (14)	14 (15)	
ECOG 3	2 (13)	1 (1)	3 (3)	
ECOG 4	0	0	0	
Toxicity of ≥ grade 3 (CTCAE 5.0), n (%)	CRT only (n=18)	CRT + D (n=83)	All patients (n = 101)	P-value
Neutropenia	4 (22)	10 (12)	14 (14)	0.27
Pneumonitis	1 (6)	6 (7)	7 (7)	1
Pneumonia	2 (11)	4 (5)	6 (6)	0.29
Oesophagitis	2 (11)	2 (2)	4 (4)	0.15
Renal failure	2 (11)	2 (2)	4 (4)	0.15
Other	6 (33)	16 (19)	22 (22)	0.48
Sum	17 (94)	40 (48)	57 (56)	0.05

CRT + D: Patients receiving durvalumab after CRT. Others include: pneumothorax, pleural effusion, confusion, encephalitis, osteomyelitis, rash, anorexia, hyperglycemia, nausea, hemoptysis, dyspnea, infection of unknown origin, atrial fibrillation, cerebral thrombosis and hypocalcemia.

The predominant grade ≥ 3 toxicities registered were neutropenia, pneumonitis, pneumonia, esophagitis and renal failure. No grade 4 toxicity was recorded. One patient in the group receiving durvalumab died of a cerebral thrombosis during treatment with durvalumab. No statistically significant differences were detected in individual toxicities between the two patient groups. However, cumulative toxicity was significantly higher in the CRT only group (**Table 3**). Among the 18 patients who did not receive durvalumab, a total of 17 grade ≥ 3 toxicities (94%) were reported. In the 83 patients receiving consolidation immunotherapy, a total of 40 grade ≥ 3 toxicities (48%) were reported.

Only three patients did not receive durvalumab due to pneumonitis or esophagitis. Factors associated with risk of developing pneumonitis and esophagitis are listed in **Supplementary Tables 1**, **2** respectively. Of the 17 patients with diabetes in this study, 11.1% developed pneumonitis, compared to 6% of the patients without diabetes (non-significant).

Logistical regression and multivariable analysis computing factors as age, diabetes, daily smoking, pre-treatment ECOG status and disease stage did not show any statistically significant correlation in determining whether a patient started durvalumab or not.

### 3.4 Biochemical parameters

Laboratory data before and after CRT are shown in **Table 4**. The laboratory data examined were hemoglobin (Hb), lactate dehydrogenase (LDH), c-reactive protein (CRP) and leukocytes. LDH before CRT was significantly higher in the group who received CRT only (p-value = 0.003) compared with the group that startet durvalumab treatment. There was no correlation between pre-CRT LDH and PTV as a measure of tumor volume. Both groups experienced a significant drop in Hb levels during CRT. The group who received CRT only had a mean fall from 13.2 g/dl to 10.8 g/dl (p-value = 0.002). The group who received durvalumab had a mean fall in Hb levels from 13.3 g/dl to 11.3 g/dl (p-value < 0.001). In addition, there was a significant relationship between low hemoglobin levels prior to CRT and disease progression after CRT. Patients with progressive disease had a mean Hb before CRT of 11.4 g/dl, while patients not progressing had a mean Hb before CRT of 13.4 g/dl (p-value = 0.040). Levels of CRP after, but not prior to CRT, were significantly higher in the group who received CRT only (p-value = 0.05). There was a significant relationship between high CRP levels prior to CRT and disease progression. In patients with progressive disease, the mean CRP at baseline was 77.4 mg/l, compared to 20.8 mg/l in patients with no progressive disease (p-value = 0.015). The patients who developed pneumonitis and pneumonia had significantly higher CRP levels after CRT than those who did not develop pneumonitis or pneumonia (p-value = 0.01 and p-value < 0.001).

**Table 4:** Laboratory data before and after CRT.

Laboratory data	CRT only (n = 18)	CRT + D (n = 83)	All patients (n = 101)	P-value
Before chemoradiotherapy, mean				
Hb, g/dl	13.2	13.3	13.3	0.75
LDH, U/l	218.6	185.6	191.6	0.003
CRP, mg/l	30.3	23.1	24.4	0.51
Leukocytes, x 10^9^/l	10.1	9.6	9.8	0.61
After chemoradiotherapy, mean				
Hb, g/dl	10.8	11.3	11.2	0.55
LDH, U/l	209	186.5	190.3	0.15
CRP, mg/l	43.5	21.4	25.5	0.05
Leukocytes, x 10^9^/l	4.1	3.9	3.9	0.85

CRT + D: Patients receiving durvalumab after CRT.

## 4 Discussion

In our trial, 18 of 101 patients (18%) did not receive durvalumab after CRT. Patients who did not start immunotherapy had a significantly higher LDH at baseline and a significantly higher CRP, cumulative toxicity and ECOG-status after CRT. Increased toxicity and poor performance status seem to limit completion of planned treatment. The median time from end of CRT to start of durvalumab was 31 days, with a significant difference between patients enrolled in 2017-2018 (median time 75 days) and patients included in 2020-2021 (median time 21 days). The delayed onset of immunotherapy in the first years was mainly due to unawareness of the benefit of early initiation of durvalumab in this setting leading to local routines for starting durvalumab about 3 months after completion of CRT. Exploratory analysis from the PACIFIC trial highlighted improved survival in patients starting durvalumab within the first two weeks after completing radiotherapy (13). Increased time after completion of CRT may be necessary for toxicities to resolve. We would expect the proportion of patients not starting consolidation therapy in our trial to be even higher if we had applied a stricter time limit from end of CRT to start durvalumab, as the added time in a number of cases enabled recovery from chemoradiation toxicity.

### 4.1 Pulmonary toxicity

Pulmonary toxicity after CRT was the main reason for not starting durvalumab for 4 patients. Pneumonitis after definitive CRT for NSCLC is associated with significant morbidity and occasionally mortality (14), and usually occurs about 4-12 weeks after completion of radiotherapy (15). Immunotherapy can also cause pneumonitis, and a study of pneumonitis after CRT and consolidation durvalumab reported highest incidence of pneumonitis 3-6 months after CRT (16). As we focused on the identification of factors preventing onset of durvalumab treatment, we recorded acute pneumonitis occurring within 3 months after CRT.

In our study, the incidence of grade ≥ 3 pneumonitis was not significantly different between the patients who received durvalumab and those who did not (7% and 6% respectively), implying that radiation-induced pneumonitis was not a major reason for not starting durvalumab. In line with previous studies, we found a non-significant trend towards higher incidence of pneumonitis in patients with diabetes (17).

The incidence of pneumonitis was significantly correlated to MLD and lung V20. This is in accordance with the QUANTEC (Quantitative analysis of normal tissue effects in the clinic) data that estimates that 10-20% of patients with MLD between 13-20 Gy will develop symptomatic pneumonitis (18). In the present study, the MLD was 17.4 Gy in the patients that developed pneumonitis while the group that did not develop pneumonitis had a MLD of 12.5 Gy (**Table 4**). Seven patients experienced pneumonitis and only one of these were in the group that did not receive durvalumab. According to the review from Marks et al., the risk of developing pneumonitis is dependent on MLD, but there is no sharp dose threshold below which there is no risk (19). However, their data seems to indicate that an MLD of 17.4 Gy carries a risk of developing radiation pneumonitis of about 16%, while an MLD of 12.5 Gy carries a risk of about 9%. The QUANTEC data (18) must nevertheless be interpreted with caution as treatment techniques have changed and with introduction of the VMAT technique, a greater portion of the lung will receive a low dose of radiation that may also influence the risk of developing pneumonitis. In addition, concomitant chemotherapy also increases the risk (20).

New radiation techniques give less pneumonitis. Patients treated with cisplatin and etoposide concomitant with radiotherapy from 31 studies were reviewed by Steuer et al. They reported a 12% incidence of grade 3-4 pneumonitis (21) which is a much higher rate than observed in the present study (7%). However, the review included studies from 1990 until 2015. Most likely, several of the studies included in the review were based on older radiation techniques. A recent meta-analysis performed by Kuang et al. found the incidence of radiation-related pneumonitis grade 3-5 to be 7.85% [95% CI 4.08-13.10] in observational studies from 2014 to 2020 using radical radiotherapy and platinum-based doublet chemotherapy in stage III NSCLC (22).

Liang et al. conducted a multicenter randomized phase III trial with CRT for unresectable stage III NSCLC (23). Radiotherapy was administered to 60-66 Gy with concomitant cisplatin and etoposide in one study arm. They utilized a simplified IMRT with a mean MLD of 15.8 Gy and a mean lung V20 of 27%, resulting in only 3% grade 3 pneumonitis. This is less than expected from the QUANTEC data (18) and less than seen in our study. Both the MLD (12.9 Gy) and lung V20 (22.5%) was higher in our study. The lower lung doses in Liang et al. may be due to their stricter dose constraints or smaller tumor volumes. Nevertheless, the patients who developed pneumonitis in the present study received an MLD of 17.4 Gy and a V20 of 35.9% which is only slightly higher than our local dose volume constraints. As pneumonitis grade ≥ 3 may influence the patients performance status and treatment plan compliance, more restrictive lung dose volume constraints may be considered. On the other hand, to improve survival, patients with large volume disease may also be offered curative treatment. The risk of toxicity must thus be balanced against the probability of improved survival.

### 4.2 Non-pulmonary toxicity

Non-pulmonary toxicity after CRT was the main reason for not starting durvaluamb for 4 patients. Esophagitis is one of the main severe toxicities during CRT in unresectable stage III NSCLC. On average 20-30% of patients will experience grade 3-4 acute esophagitis requiring tube- or intravenous feeding (24). In our trial, two patients did not receive durvalumab due to esophagitis grade 3. However, the incidence of grade 3 esophagitis was not significantly different between the group who started durvalumab and those who did not, indicating that esophagitis may not be a major reason preventing the onset of durvalumab.

A previous study found that radiation induced esophagitis was the reason why six percent of the patients interrupted radiotherapy, causing a prolonged radiation treatment time (25) which may have a detrimental effect on radiation response. Another study reported a 20% occurrence of grade 3 esophagitis (23). This was much higher than in the present study (4%) despite lower doses of chemotherapy (cisplatin was administrated with 50 mg/m^2^ on day 1 and 8 every 4 weeks, and etoposide 50 mg/m^2^ on day 1-5). Their excess esophageal toxicity may be caused by their use of simplified IMRT leading to a higher radiation dose to neighboring organs. In the review by Steuer et al. including 31 studies, the median cisplatin dose was 50 mg/m2 and etoposide 50 mg/m2 and the median radiation dose to the target volume was 63 Gy. They reported a 23% incidence of grade 3-4 esophagitis (21). This may indicate that the influence of chemotherapy is less important than the contribution from radiotherapy. The radiation techniques utilized in the studies for the review, were most likely different to more modern techniques as the review included studies from 1997 until 2013. The radiation dose to the esophagus may thus have been higher than in the present study,

Based on the QUANTEC data a mean radiation dose of < 34 Gy to the whole of the esophagus may result in a 5-20% risk for developing ≥ grade 3 esophagitis (18). Accordingly, the mean esophageal radiation dose to the whole esophagus, is required to be less than 34 Gy with our local dose volume constraints. In the present study the mean esophagus dose was 37.4 Gy in the patients who developed grade 3 esophagitis. Even though the incidence of grade ≥ 3 esophagitis in the present study was low, further improvements may be achieved by strict adherence to specified dose volume constraints. Applying additional dose constraints to a smaller volume of esophagus may also be considered. Zhang et al. reported risk factors for radiation induced acute esophagitis in patients with NSCLC treated with CRT (26). They found that V50 and concomitant chemotherapy correlated with grade 3 toxicity and duration. Others have found a significant correlation between V50 and V55 and esophagitis (27). Shrinking radiation field techniques according to tumor response has also been applied with a 12% incidence of grade 3 esophagitis (28). However, the incidence was still higher than in the present study.

### 4.3 Biochemical parameters

Biochemical parameters are signs of underlying processes and not themselves reasons for not starting durvalumab or targets for preventive measures. They may, however, help us understand the difference in disease and/or treatment response between the two groups.

Pre-CRT LDH was significantly higher in the CRT only group (p-value = 0.003). LDH is a known poor prognostic marker in NSCLC and a predictor of treatment resistance including reduced effect of platinum-based chemotherapy (29, 30). There was no correlation between PTV and pre-CRT LDH. This may indicate that tumor volume dose not contribute to a significant difference in pre-CRT LDH among the study patients and that it rather reflects biological processes in the tumor and confirms the poor prognosis.

CRP at baseline was found to be significantly higher among the patients who had progressive disease compared to those who did not have progressive disease (p-value = 0.015). The prognostic role of CRP for patients with NSCLC is not clear. CRP has been found associated with better prognosis in patients with advanced NSCLC treated with chemotherapy, but also with poor survival in both early and late stage disease (31–33).

After CRT, the patients who did not start consolidation treatment had on average double the CRP-values found in the group that received durvalumab (p-value = 0.05). Since there was no significant difference in leukocyte counts between the groups, the differences in CRP are most likely not due to infections and could rather be due to side effects of the treatment or the disease itself. It is well known that CRP levels increase during radiotherapy (34). Chemotherapy, however, has been found to reduce the CRP levels in patients with NSCLC and a reduction is associated with response to treatment (35, 36). These patients received both chemotherapy and radiotherapy. The difference between the groups may both represent a difference in response/tumor aggressiveness and in side effects/inflammation. The patients who developed pneumonitis and pneumonia had significantly higher CRP values after CRT than those who did not develop pneumonitis or pneumonia (p-value = 0.01 and p-value < 0.001) indicating that some of the difference between the CRP levels in the two groups is caused by adverse events in the lung.

Low hemoglobin (Hb) has been reported to be associated with reduced survival after CRT for NSCLC. Crvenkova et al. found that patients with hemoglobin levels ≤ 12 g/dl, had a worse survival (2). In the present study, disease progression was significantly associated with lower baseline Hb levels (p-value = 0.04). This is in line with previous studies showing that low baseline Hb levels are associated with poor response to CRT in patients with anal cancer (37). While both groups of patients in our study experienced a significant drop in Hb level during CRT, there was a trend toward a greater decrease in patients who did not start durvalumab. Lower Hb levels may influence the response to radiotherapy as tumors may be hypoxic and reoxygenation is important for radiotherapy response (38). Similarly, tumor cell repopulation increases during the course of radiotherapy and Hb levels during the latter part of radiotherapy may be important to ensure the availability of free radicals causing permanent DNA damage and thus tumor cell death (38). Myelotoxicity following chemotherapy and radiotherapy to marrow-containing bone, may contribute to the drop in Hb level after CRT (39). Infections and inflammation can also contribute to the development of anemia (40). Questions have been raised as to whether transfusion of red blood cells before and during radical radiotherapy might improve outcomes such as locoregional control and overall survival, but there is currently no evidence supporting transfusion outside of conventional thresholds in this setting (41).

### 4.4 Possible preventive measures

The interest of prehabilitation is increasing in cancer treatment. Prehabilitation encompasses the health care given prior to medical or surgical interventions. The aims of prehabilitation may thus be to ensure more patients to complete treatment, reduce the amount and severity of complications, increase the level of function after treatment and improve quality of life (42). A multimodal and multidisciplinary approach is needed to secure the best possible prehabilitation tailored to each patient. This study may help elucidate possible points of interest to tailor prehabilitation. Although not significant, our data showed a trend of lower DLCO in the CRT only group. Santus et al. reported improved DLCO after habilitation (43). Lung physiotherapy may reduce the risk of lung infections and secondary complications. Similarly, it is possible that focus on nutrition and exercise may improve patients’ performance status after CRT. Borghetti et al. found a home-based rehabilitation program consisting of endurance and resistance training to significantly improve exercise capacity and prevent physiological impairment of quality of life in patients undergoing radio(chemo)therapy for locally advanced lung cancer (44). There are still uncertainties with the effect of prehabilitation in this group of patients and more research is needed. However, targeted goals and personalized programs customized to the preferences and possibilities of the patients may increase the likelihood of success (45).

As long as the treatment intent is curative, reducing doses to the target volume is not an option. In order to minimize the risk of treatment abruption, reducing the radiation doses to the organs at risk remains important, and planning techniques are constantly improved to accommodate this need. From our data, limiting the doses to the lung and to the esophagus may be of highest priority, while reducing the overall level of toxicity is important in itself. Prehabilitation, including exercise training, nutritional assessment and smoking cessation starting as soon as possible may improve the pre-treatment and exercise capacity through improved DLCO, prevent decline in performance status and ensure optimal conditions for tumor radiation response. Prospective intervention trials are needed.

### 4.5 Study limitations

The present study is retrospective and enrolled a relatively small number of patients (n=101) which influences the power of this study. The number of patients was restricted by the patient populations enrolled in prior programs/trials. As this is an exploratory study, we emphasize trends over statistical significance and multiple testing has not been done. The study is hypothesis-generating rather than concluding andhe results should be validated in larger, prospective studies.

## 5 Conclusions

An 18% treatment plan disruption rate, as shown in this study, seems rather high and elucidation of factors associated with worse outcome may help future patient treatment selection. The group of patients that did not receive consolidation durvalumab, had higher LDH and lower DLCO prior to CRT and worse performance status, lower Hb and higher CRP after CRT compared with the group of patients that received durvalumab after CRT. While no specific toxicity was associated with not starting durvalumab, the CRT only group experienced significantly higher cumulative toxicity. Prehabilitation including physical activity may improve DLCO. Optimized nutritionand prehabilitation should be explored for effect on post-CRT performance status.

The present study confirmed that radiation doses were associated with the development of pneumonitis and esophagitis of grade 3 after CRT. Limiting the doses to these organs is important. Furthermore, with newer radiation techniques and concomitant chemotherapy, there is a need to establish new dose-volume constraints. Patients with diabetes mellitus may have an increased risk for developing toxicity as pneumonitis, but this needs to be elucidated in a larger study population. Further research on toxicities and the effects of preventive measures such as prehabilitation may reduce the number of patients not completing planned treatment and thereby improve survival for these patients.

## Data availability statement

The raw data supporting the conclusions of this article will be made available by the authors, without undue reservation.

## Ethics statement

The studies involving human participants were reviewed and approved by Regional Ethical Comittee South-East no 22/426980. The patients/participants provided their written informed consent to participate in each substudy.

## Author contributions

CL: Formal analysis, investigation, writing - original draft. HH: Data analysis and reviewed and approved of the final manuscript. ÅH: Conceptualization and reviewed and approved of the final manuscript and VH: Conceptualization, data analysis and reviewed and approved of the final manuscript. All authors contributed to the article and approved the submitted version.
