# Supplementary material for: Factors associated with failure to start consolidation durvalumab after definitive chemoradiation for locally advanced NSCLC
Source: Front Oncol. 2023 Jul 5;13:1217424. doi: 10.3389/fonc.2023.1217424 (PMC10354813; doi:10.3389/fonc.2023.1217424)
Supplement: Supplementary file 1 [file DataSheet_1.docx]

Supplementary Material

Factors associated with failure to start adjuvant durvalumab after definitive chemoradiation for locally advanced NSCLC

Christian Wilhelm Langberg, Henrik Horndalsveen, Åslaug Helland, Vilde Drageset Haakensen*

*** Correspondence:**Vilde Drageset Haakensen: [vilde.haakensen@gmail.com](mailto:vilde.haakensen@gmail.com), [vdd@ous-hf.no](mailto:vdd@ous-hf.no)

# Supplementary Figure 1

**Supplementary Figure 1**: Study flow chart

# Supplementary Table 1

| Pneumonitis associations | Pneumonitis  (n = 7) | No pneumonitis  (n = 94) | P-value |
| --- | --- | --- | --- |
| Age |  |  | 0.21 |
| Age, mean | 70 | 66.2 |  |
| Stage, n |  |  | 0.17 |
| Stage IIB | 0 | 7 |  |
| Stage IIIA | 3 | 49 |  |
| Stage IIIB | 2 | 34 |  |
| Stage IIIC | 2 | 4 |  |
| Planning target volume, cm^3^, mean |  |  |  |
| PTV, (range) | 539.8 (274-824) | 419 (70-1197) | 0.26 |
| Mean lung dose, Gy, mean |  |  |  |
| MLD, (range) | 17.4 (14-20) | 12.5 (3-21) | 0.01 |
| Lung V20, %, mean |  |  |  |
| Lung V20, (range) | 29.9 (22-35) | 21.9 (5-35) | 0.019 |
| Total radiation dose, Gy, mean |  |  |  |
| Total dose, (range) | 64.3 (60-66) | 64.9 (60-66) | 0.52 |
| Diabetes, n |  |  | 0.34 |
| Diabetes | 2 | 15 |  |
| No diabetes | 5 | 79 |  |
| Lung function before CRT |  |  |  |
| FEV1, l | 2.8 | 2.1 | 0.08 |
| FEV1, % | 91.7 | 75.8 | 0.12 |
| DLCO, % | 63.5 | 67.6 | 0.59 |
| Smoking status, n |  |  | 0.77 |
| Previously smoked daily | 5 | 68 |  |
| Daily smoker | 2 | 21 |  |
| Never smoked | 0 | 5 |  |
| Neutropenia, n |  |  | 1 |
| Neutropenia | 1 | 13 |  |
| No neutropenia | 6 | 81 |  |

**Supplementary Table 1**: Factors associated with pneumonitis

# Supplementary Table 2

| Esophagitis associations | Esophagitis  (n = 4) | No esophagitis  (n = 97) | P-value |
| --- | --- | --- | --- |
| Mean oesophagus dose, Gy, mean |  |  |  |
| Mean oesophagus dose, (range) | 37.4 (22-50) | 19.5 (4-61) | <0.001 |
| Neutropenia, n |  |  | 0.09 |
| Neutropenia | 2 | 12 |  |
| No neutropenia | 2 | 85 |  |
| Age, years, mean |  |  |  |
| Age | 64 | 66.6 | 0.52 |
| Diabetes, n |  |  | 0.53 |
| Diabetes | 1 | 16 |  |
| No diabetes | 3 | 81 |  |
| Smoking status, n |  |  | 1 |
| Previously smoked daily | 3 | 70 |  |
| Daily smoker | 1 | 22 |  |
| Never smoked | 0 | 5 |  |
| Stage, n |  |  | 1 |
| Stage IIB | 0 | 7 |  |
| Stage IIIA | 2 | 50 |  |
| Stage IIIB | 2 | 34 |  |
| Stage IIIC | 0 | 6 |  |

**Supplementary Table 2**: Factors associated with esophagitis
